# Clinical Validation of a Deep Learning-Based 2D Ultrasound Steatosis Algorithm: Cutoff Transferability, Scanner Generalizability, and Comparison with FibroScan

**DOI:** 10.3390/diagnostics16020267

**Published:** 2026-01-14

**Authors:** Jennifer Tai, Tse-Hwa Hsu, Cheng-Jen Chen, Ming-Ling Chang, Chihung Lin, Shiu-Feng Huang, Le Lu, Adam P. Harrison, Dar-In Tai

**Affiliations:** 1 Department of Gastroenterology and Hepatology, Chang Gung Memorial Hospital at Linkou, Taoyuan 333423, Taiwan; jennifertai112@gmail.com (J.T.); echohsuth45@gmail.com (T.-H.H.); k85731@cgmh.org.tw (C.-J.C.); mlchang8210@gmail.com (M.-L.C.); 2 Center for Artificial Intelligence in Medicine, Chang Gung Memorial Hospital, Taoyuan 333, Taiwan; lin3031@gmail.com; 3 Institute of Molecular and Genomic Medicine, National Health Research Institutes, Zhunan, Miaoli 35053, Taiwan; sfhuang@nhri.org.tw; 4 DAMO Academy, Alibaba Group, New York, NY 10014, USA; tiger.lelu@gmail.com; 5 Research Division, Riverain Technologies, Miamisburg, OH 45342, USA

**Keywords:** deep learning, 2D ultrasound, quantitative ultrasound, liver steatosis, liver histology, controlled attenuation parameter, diagnostic performance

## Abstract

**Background:** Liver steatosis assessment by 2D ultrasound is widely used but remains subjective. We previously developed a deep learning (DL) algorithm for objective steatosis quantification. This study aimed to (1) establish histology-based cutoffs, (2) evaluate their transferability across different imaging views, and (3) validate performance on a new scanner not included in training. **Methods:** We retrospectively analyzed 588 ultrasound studies from 457 histology-proven cases and prospectively collected paired scans using a new scanner (Philips Affiniti 70). Images from right intercostal, left hepatic lobe, and subcostal views were processed with the DL algorithm, and mean values from 3–5 images per view were correlated with histology. **Results:** Across three views, the DL algorithm achieved AUROCs of 0.891–0.936 across steatosis grades, consistently outperforming FibroScan’s controlled attenuation parameter (0.840–0.905), especially in moderate-to-severe steatosis (*p* < 0.001). Cutoffs established from right intercostal images (N = 565) were applied to images from left hepatic lobe (N = 464) and subcostal views (N = 341), yielding accuracies of 0.792–0.850. On Affiniti 70 images, AUROCs remained high (0.838–0.896), supporting scanner generalizability. **Conclusions:** The DL algorithm provides accurate, view-independent steatosis grading across different ultrasound scanners and outperforms CAP, supporting its real-world use for objective, reproducible quantification.

## 1. Introduction

Metabolic dysfunction-associated steatotic liver disease (MASLD) has become the leading cause of diffuse liver disease, following the global success of hepatitis B vaccination programs and the widespread use of direct-acting antiviral therapies for hepatitis C virus (HCV) [1,2]. Excess hepatic fat accumulation can lead to inflammation, fibrosis, and, in severe cases, hepatocellular carcinoma (HCC) [3,4,5]. MASLD is strongly associated with obesity and diabetes mellitus, with steatosis severity correlating with higher risks of hepatic and extrahepatic complications [2,3]. However, a substantial proportion (7–20%) of individuals with liver steatosis are non-obese, highlighting the need for accurate, non-invasive diagnostic methods for broader patient populations [6,7,8].

The current gold standards for steatosis quantification are liver biopsy and magnetic resonance imaging proton density fat fraction (MRI-PDFF) [9,10]. Biopsy is invasive, subject to sampling bias, and impractical for routine screening, while MRI-PDFF is expensive and not widely accessible. Although 2D ultrasound is widely available and inexpensive, conventional assessment of steatosis remains qualitative and subjective, limiting clinical utility.

Several quantitative ultrasound (QUS) methods [11,12,13,14,15], including FibroScan’s controlled attenuation parameter (CAP) [16,17], have been developed and clinically adopted for steatosis assessment. However, QUS often requires specialized equipment, predefined regions of interest, and standardized scanning protocols, limiting cross-system comparabilityImportantly, these QUS seem unable to differentiate between grade 2 and grade 3 steatosis well [14]. Deep learning (DL) applied to standard 2D ultrasound offers a promising alternative [18,19,20,21,22,23,24], enabling automated and objective analysis of entire ultrasound images without manual ROI selection, and allowing retrospective application to existing datasets for both clinical practice and research. In several recent studies, using deep leaning models with different scanners and views [18,22,24]. the AUC for quantification of steatosis may reach around 0.9 in different stages of steatosis. This approach successfully changes the conventional ultrasound from a subjective diagnosis to quantitative measurement.

In our previous work, we developed a DL algorithm that was trained by more than 200 thousand 2D-US images using ResNet18 as backbone, which accurately quantified steatosis across multiple scanners and views in histology-proven cases [18]. However, the diagnostic cutoff values were not well established. In this study, we increased the number of histology-proven cases and excluded those used in the previous training process as well as US images obtained from old scanners

## 2. Materials and Methods

This study was approved by the Institutional Review Board (IRB) of the Chang Gung Medical Foundation (CGMH IRB No. 201801283B0 and 202200758B0).

### 2.1. Patients

FibroScan (Echosens, Paris, France) has been available at Chang Gung Memorial Hospital, Linkou Medical Center, since 2015. As part of the center’s policy, FibroScan is routinely performed for all patients undergoing liver histology studies.

We prospectively enrolled consecutive patients who underwent both liver biopsy and FibroScan between April 2015 and April 2025. The primary indications for liver biopsy included evaluation of abnormal liver function tests, assessment of liver fibrosis, and diagnosis of liver tumors. All patients received comprehensive virological and immunological workups. Laboratory tests—including platelet count, prothrombin time, and transaminase levels—were performed within one week of the liver biopsy.

**Figure 1:**
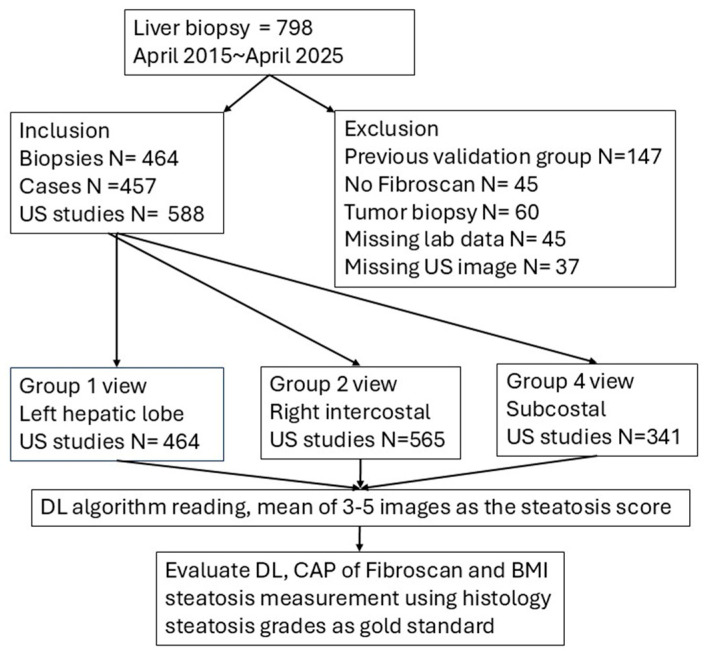
Flowchart. We prospectively enrolled consecutive patients who underwent both liver biopsy and FibroScan between April 2015 and April 2025. All patients received comprehensive virological and immunological workups. Laboratory tests—including platelet count, prothrombin time, and transaminase levels—were performed within one week of the liver biopsy. Patients were excluded if they had been previously included in the validation group, had tumor-only biopsy specimens, and incomplete studies. After inclusion, the images were read by DL steatosis algorithm according to their views. A mean of 3–5 measurements will become the final result.

Patients were excluded if they had been previously included in the validation group, lacked FibroScan measurements, had tumor-only biopsy specimens, incomplete laboratory data, or insufficient 2D ultrasound imaging obtained within three months of the liver biopsy (Figure 1).

### 2.2. 2D Ultrasound

Since 2011, we have been providing free acoustic radiation force impulse (ARFI) elastography using the Acuson S2000 system (Siemens Healthcare, Erlangen, Germany) for all patients undergoing liver biopsy at our center [25]. A 2D ultrasound examination was also performed using the same system with a convex probe (4C1) during the same imaging session.

All participants were instructed to fast for at least four hours before the ultrasound examination. If 2D ultrasound images from the Siemens system were not available, images from other ultrasound systems obtained within three months of the liver biopsy were retrospectively collected.

### 2.3. Prospective Study on a New 2D Ultrasound Scanner

Additionally, to evaluate the generalizability of the DL steatosis algorithm across different ultrasound systems, we conducted a prospective study using paired 2D ultrasound examinations. Each patient underwent scanning with both the Siemens Acuson S2000 and the Philips Affiniti 70 (Seattle, WA, USA) system, equipped with a 3.5 MHz transabdominal probe. Notably, the Affiniti 70 was not included in the algorithm’s training dataset. Both 2D ultrasound studies were performed on the same day and within 1 month of liver biopsy.

FibroScan studies were funded through research grants and provided to all patients undergoing liver biopsy. Measurements were performed according to the manufacturer’s specifications and were primarily conducted by a single experienced technician (T-H) within one month of liver biopsy. On rare occasions, other trained personnel performed the measurements in her absence. The CAP was assessed using ten validated measurements, following the same criteria applied for liver stiffness evaluation. The final CAP value was calculated as the median of these individual measurements.

### 2.4. Image Selection and Classification

For the available 2D ultrasound studies, images were excluded if they were non-hepatic, contained only a limited portion of liver parenchyma, had poor image quality, or displayed unidentified scanning views, as determined by two reviewers (T-H and D-T).

No additional adjustments were made to the selected images. We assume that the examiners sought optimal scanning to aid diagnosis. Using the same images that had already been assessed by the US operators during their clinical examinations, our primary goal was to transition liver steatosis evaluation from a subjective assessment to an objective diagnosis.

The remaining images were then categorized into four scanning views, as defined in our previous study [18]:

• Left hepatic lobe view (G1);
• Right intercostal view (G2; right kidney excluded);
• Liver-kidney contrast view (G3);
• Subcostal view (G4; right kidney excluded).

Our previous studies demonstrated that the G3 view had lower repeatability and accuracy [18,26], and it was therefore excluded from current analysis. In addition, prior findings suggest that at least 3–5 images per view are necessary for reliable quantification; thus, scanning view groups with fewer than three images were also excluded.

### 2.5. DL Steatosis Algorithm and Image Reading

The DL steatosis algorithm was developed in our previous study [18]. It was trained and validated on 2899 patients and 228,075 ultrasound images acquired via 13 different scanners from patients undergoing elastography. The algorithm is based on ResNet18 [27] with images processed for de-identification, segmentation, normalized brightness and size, and rotated at random angles between 0 and 10 degrees. After training, it produces a continuous steatosis severity score from 0 to 1. More information about the training process—including the use of ordinal regression loss, tuned hyper-parameters, and image augmentation—can be found in the Supplementary Material of our previous work [18].

In this study, the DL steatosis algorithm was applied to the selected and annotated images. The final DL steatosis score for each patient was calculated as the mean score from 3–5 images per view group. The correlation between different views was more than 0.8 [18,26].

### 2.6. Histology Grading

To minimize inter-observer variability, liver histology was evaluated by an experienced pathologist (S-H), who was blinded to the present study. Steatosis severity was graded based on the hepatic fat cell fraction, following the classification by Kleiner et al. [28]:

• Normal: <5%;
• Mild: ≥5% and <33%;
• Moderate: ≥33% and <66%;
• Severe: ≥66%.

Liver fibrosis was staged semi-quantitatively from 0 to 4 using either the Kleiner et al. or METAVIR scoring systems [29]. In cases of discordance between the two classifications, the higher stage was assigned.

### 2.7. Statistical Analysis

Patient characteristics were summarized as frequencies and percentages for categorical variables, and as mean ± standard deviation (SD) or standard error of the mean (SEM) for continuous variables, as appropriate. Categorical variables were compared using the chi-square test or Fisher’s exact test, while continuous variables were analyzed using one-way ANOVA. The Mann–Whitney U tests were performed to compare DL steatosis scores between two grades, and partial correlation analysis was performed to assess the impact of fibrosis on DL steatosis measurements.

Model diagnostic performance was evaluated via receiver operating characteristic (ROC) curves, with the area under the ROC curve (AUROC) serving as the primary metric. Optimal cutoffs were determined using the Youden index to balance sensitivity and specificity. Correlation analyses were performed to investigate differences in DL steatosis scores among various scanning views. DeLong’s test was applied to compare AUROCs. In a portion of the ultrasound study, two separate scanners were utilized for identical analyses. Furthermore, Obuchowski’s non-parametric ROC method was applied to the G2 views to account for correlated measurements within the same patients.

Statistical analyses were performed using SPSS (v22), with R for non-parametric ROC studies. Additional calculations were performed using Microsoft Excel, and graphs, including ROC curves, were created in Stata/MP 14.0 for Mac. Statistical significance was defined as *p* < 0.05.

## 3. Results

### 3.1. Patient Demographics

A total of 457 patients with 464 liver biopsy studies (including six patients who underwent more than one biopsy) and 588 2D ultrasound studies were included in the final analysis (Figure 1). Of these patients, 55 were seropositive for hepatitis B surface antigen (HBV) and 12 were seropositive for hepatitis C virus (HCV) antibodies. The remaining 390 patients were seronegative for both HBsAg and anti-HCV and were classified as the non-HBV non-HCV (NBNC) group. Patients with HBV or HCV were categorized into the viral hepatitis group.

The demographic and clinical characteristics of the two groups are summarized in Table 1. Compared to the NBNC group, patients with viral hepatitis were more often male (*p* = 0.046), and had a significantly older age (55.3 ± 11.0 vs. 49.2 ± 14.0 years, *p* < 0.001), lower platelet counts (*p* < 0.001), lower total cholesterol levels (*p* = 0.003), and higher PT-INR (*p* = 0.006). Regarding associated etiologies, the prevalence of autoimmune hepatitis, primary biliary cholangitis, and acute hepatitis did not differ significantly between the NBNC and viral hepatitis groups. In contrast, the distributions of hepatic steatosis and fibrosis grades were significantly different between the groups (both *p* < 0.001). Severe steatosis (S3) was more prevalent in the NBNC group (36.9% vs. 14.9%), while advanced fibrosis (F4) was more common in the viral hepatitis group (26.9% vs. 6.9%).

### 3.2. Ultrasound Scanner Models

Six different ultrasound scanner models were used in this study (Table 2). The primary scanner was the Siemens Acuson S2000, which accounted for 439 studies (74.7%). This predominance was primarily due to the provision of free elastography for patients undergoing liver biopsy, with the Siemens Acuson S2000 being used for both elastography and 2D ultrasound in the same examination room. This setup facilitated the convenient completion of both studies during a single visit.

Among the six scanners, the Philips Affiniti 70 was the most recently introduced and was not included in the training dataset. Its performance was compared to that of the Siemens Acuson S2000, using examinations performed on the same day.

### 3.3. Diagnostic Performance Among Different Modalities

**Figure 2:**
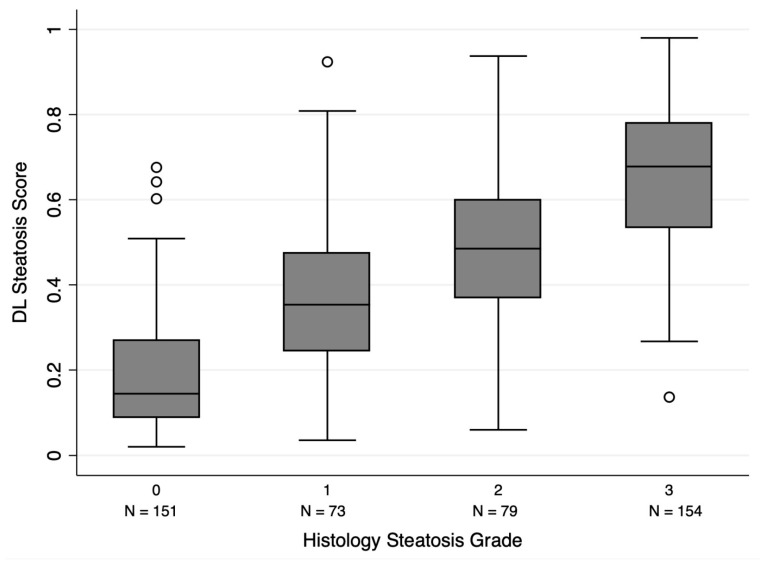
DL algorithm-derived steatosis scores stratified by histopathological steatosis grade. DL scores demonstrated a significant stepwise increase across grades, with all pairwise comparisons between adjacent groups yielding *p* < 0.001 (Mann–Whitney U test).

The distribution of DL steatosis scores across different histological steatosis grades is shown in Figure 2. Significant differences in DL scores were observed between each pair of adjacent grades (*p* < 0.001, Mann–Whitney U test).

**Figure 3:**
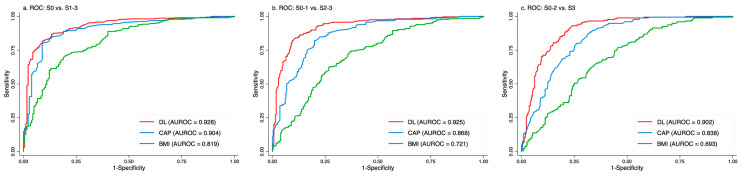
ROC curves of the DL steatosis score, FibroScan CAP, and BMI in the diagnosis of liver steatosis using the G2 (right intercostal) view: (**a**) Steatosis grades 0 vs. 1–3: The DL steatosis score (AUROC = 0.929) was comparable to CAP (AUROC = 0.905, *p* = 0.131); (**b**) Steatosis grades 0–1 vs. 2–3: The DL steatosis score achieved the highest AUROC (0.927), followed by CAP (0.869) and BMI (0.722). The difference between the DL steatosis score and CAP was statistically significant (*p* < 0.001); (**c**) Steatosis grades 0–2 vs. 3: The DL steatosis score (AUROC = 0.904) significantly outperformed CAP (AUROC = 0.840, *p* < 0.001) in identifying steatosis grades 2 and 3.

Figure 3 and Table 3 present the ROC curves for DL steatosis scores, FibroScan CAP, and BMI in detecting steatosis grades using the G2 view. DL consistently achieved the highest AUROCs (0.904–0.929), followed by CAP (0.840–0.905), while BMI showed the lowest performance (0.692–0.817) across all thresholds. The ROC curves were measured using parametric and non-parametric analysis. There is no significant difference between two methods. Non-parametric ROC curves of G2 view were presented in Figure 3.

AUROCs of the DL steatosis score and CAP were compared using DeLong’s test. The *p*-values were 0.131 for grade 1 (S0 vs. S1–S3), <0.001 for grade 2 (S0–S1 vs. S2–S3), and <0.001 for grade 3 (S0–S2 vs. S3), indicating that the DL algorithm significantly outperformed CAP in detecting moderate-to-severe and severe steatosis.

Similar findings were observed in the G1 and G4 views (Table 3 and Supplementary Figure AUROCs in G1 view show similar or even better than G2 view).

### 3.4. Effect of Fibrosis on DL Steatosis Scores

Although fibrosis alters the echotexture in 2D ultrasound, DL steatosis scores remained highly correlated with histologic steatosis grade after adjustment for fibrosis (partial correlation r = 0.822, *p* < 0.001). Conversely, when controlling for steatosis grade, a negative correlation was observed between DL steatosis scores and fibrosis stage (partial correlation r = –0.281, *p* < 0.001).

### 3.5. Cutoff Values for Prediction of Histology Steatosis Grades

The G2 view is frequently used in studies to quantify liver steatosis and is often selected because it typically provides larger sample sizes suitable for developing diagnostic cutoff values. Using the G2 view, optimal DL algorithm cutoff values for histologic steatosis grades were determined by the Youden index from ROC analyses (Table 4):

• Grade 3: ≥ 0.55;
• Grade 2: ≥ 0.44;
• Grade 1: ≥ 0.26.

These G2-derived cutoffs were subsequently applied to the G1 and G4 views for comparison. Despite differences in imaging orientation, DL performance remained robust, with sensitivity, specificity, and accuracy summarized in Table 4. The DL algorithm achieved accuracies ranging from 0.792 to 0.875 across different steatosis grades and ultrasound views, supporting the transferability of cutoff values beyond the derivation view (G2).

CAP cutoff values were similarly determined as:

• Grade 3: ≥ 288 dB/m;
• Grade 2: ≥ 274 dB/m;
• Grade 1: ≥ 251 dB/m.

Across all steatosis grades, the DL steatosis score consistently demonstrated superior diagnostic performance to CAP. The difference was most evident in grade 3, where DL achieved a higher accuracy (0.825 vs. 0.750), primarily due to higher sensitivity (0.825 vs. 0.750).

The Cross Confusion Matrix: DL vs. CAP predictions by histological grades in G2 view are shown in Supplementary Table S1.

### 3.6. Evaluation of a New Scanner (Affiniti 70, Philips)

A newly introduced ultrasound scanner, the Philips Affiniti 70, was implemented in our examination room within the past year. Since this device was not included in the DL algorithm’s training dataset, we evaluated whether it could still accurately quantify hepatic steatosis using images acquired from this system.

A total of 47 patients underwent ultrasound examinations using both the Philips Affiniti 70 and Siemens Acuson S2000 scanners, in addition to FibroScan and liver biopsy. Using histology as the gold standard, ROC curves were generated for the DL steatosis scores from both scanners.

**Figure 4:**
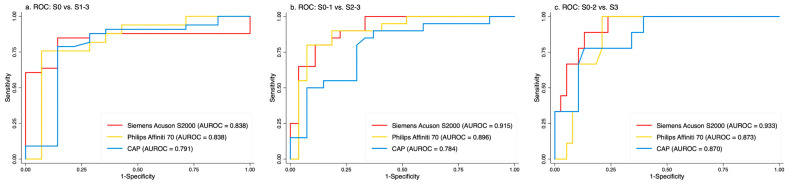
ROC curves compare DL steatosis scores from a new scanner (Philips Affiniti 70), the original training scanner (Siemens Acuson S2000), and CAP. (**a**) The DL algorithm showed similar AUROCs across all three models for grade 1 steatosis prediction; (**b**) Accuson 2000 and Affiniti 70 had similar, superior AUROCs compared to CAP for grade 2 steatosis; (**c**) All three models performed similarly for grade 3 steatosis. These results demonstrate the algorithm’s generalizability.

As shown in Figure 4, the DL steatosis scores from the Affiniti 70 yielded AUROCs comparable to those from the Acuson S2000 across all steatosis grade thresholds (e.g., 0.838 vs. 0.838 for grade 1, 0.896 vs. 0.915 for grade 2, and 0.873 vs. 0.933 for grade 3), supporting the algorithm’s generalizability across different ultrasound scanners. Notably, DL scores from both scanners consistently outperformed CAP across all steatosis grades.

Although the ROC curves showed minimal differences, the diagnostic accuracy of the Affiniti 70 was lower than that of the S2000 when using the previously established cutoff system (Supplementary Table S2). This performance can be improved by applying an Affiniti 70-specific cutoff system.

## 4. Discussion

We evaluated our DL steatosis algorithm using retrospectively collected 2D ultrasound images from 457 histology-proven cases. Based on ROC analyses, we established optimal DL steatosis score cutoffs corresponding to histologic steatosis grades (none, mild, moderate, severe) (Table 3). Our findings suggest that the DL steatosis score provides objective, image-based quantification and demonstrates superior performance compared with CAP in detecting moderate to severe steatosis (Figure 3).

Several machine learning studies have applied 2D ultrasound for diagnosing liver steatosis [23]. However, most focused on binary classification (steatosis present vs. absent), often reporting excellent AUROCs (>0.9). While binary classification is informative, histology-based grading systems [28] provide a more granular assessment of steatosis severity. DL algorithms that align with these clinical grading systems are more relevant for practice. Recent studies have explored 3- or 4-class steatosis grading schemes. Among these, studies using liver histology or MRI-PDFF as the reference standard are summarized in Table 5 [14,18,21,22,24,30]. Most reported AUROCs > 0.9 for grade 1 steatosis, with three studies [14,18,30] also achieving AUROCs > 0.9 for grades 2 or 3.

In the study by Jeon et al. [14], B-mode ultrasound images were combined with tissue attenuation imaging (TAI) and tissue scatter-distribution imaging (TSI) as training inputs. This multimodal approach outperformed models using TAI or TSI alone, achieving the highest AUROCs among the studies listed in Table 5. However, since their model relies on TAI and TSI, it is limited to Samsung ultrasound scanners. In contrast, our previous work demonstrated consistent DL performance across multiple ultrasound vendors [18], highlighting the broader applicability of our approach.

In this study, we further validated our DL algorithm on 457 histology-proven cases and compared it with CAP. For images obtained from the G2 (right intercostal) view, the AUROC of the DL score remained >0.9 across all steatosis grades (Figure 3). DeLong’s test revealed significantly superior performance of the DL steatosis score over CAP for both grade 2 (Figure 3b) and grade 3 (Figure 3c) steatosis, with *p*-values < 0.001. These results support the clinical utility of the algorithm for accurate, objective assessment of hepatic steatosis using standard B-mode ultrasound.

Our DL algorithm outperforms FibroScan’s CAP in assessing liver steatosis by leveraging diverse and comprehensive imaging features, resulting in a more holistic analysis. CAP primarily measures ultrasound attenuation, which correlates with liver fat content. However, the relationship between attenuation and steatosis severity is nonlinear, often plateauing at moderate to severe steatosis [31,32]. In contrast, 2D ultrasound-based models can analyze multiple imaging features—such as parenchymal brightness, vascular structure visibility, diaphragm clarity, and liver-kidney contrast [33]—allowing for a more accurate correlation with steatosis grading, especially in severe steatosis [14].

Among the studies in Table 5, most of the studies used single scanners, while Vianna et al. used 7 scanners24. The use of multiple scanners during training process may avoid scanner factors and focus on steatosis characteristics. Our DL algorithm was initially trained on images from 13 different ultrasound scanners in a large cohort of patients undergoing elastography [18]. The primary scanners included: Philips IU22 (45.2%), Aloka SSD 5500 (21.9%), Toshiba TUS-A300 (16.1%) and ATL HDI 5000 (14.7%). Only 1% of the training images were from a Siemens Acuson S2000 scanner. In contrast, the Siemens Acuson S2000 was the primary scanner in this study (74.7%, Table 2). Despite these differences, the DL steatosis scores correlated well with histology grades, demonstrating robust generalizability across different ultrasound scanners.

To further assess scalability, the DL algorithm was tested prospectively on the Philips Affiniti 70, a new scanner not included in training. Performance remained high and comparable to that of the Siemens Acuson S2000, reaffirming the model’s generalizability across devices (Figure 4). However, the accuracy of Affinitei 70 is relatively lower than S2000 when preset cutoff values were applied. The accuracy can be improved when a specific cutoff derived from the Affiniti 70 scanners is applied (Supplementary Table S2).

In certain clinical scenarios—such as patients with a post-right lobectomy, or those with large hepatic tumors or cysts—the standard right intercostal (G2) view may not be obtainable, necessitating alternative views for assessment. In previous reports, Vianna et al. [24] and Byra et al. [22] also use views other than RLL. Therefore, we also evaluated the DL algorithm’s performance using:

• G1 view (left hepatic lobe): AUROCs of 0.915–0.936, significantly outperforming CAP (0.842–0.901; *p* < 0.001 for grades 2–3; Supplementary Figure S1).
• G4 view (subcostal): AUROCs of 0.891–0.914, numerically higher than CAP (0.865–0.883), but the differences were not statistically significant (*p* = 0.133–0.372; Supplementary Figure S2).

The AUROC results in the G1 view were comparable to, or even better than, those in the G2 view. These findings indicate that the DL algorithm can effectively quantify liver steatosis across multiple imaging views, offering greater flexibility in clinical practice.

Unlike QUS and elastography, which require placement of a region-of-interest (ROI) in the right hepatic lobe, our DL model does not require manual ROI selection, facilitating integration into routine ultrasound workflows. Moreover, G2-derived cutoffs transferred well to G1 and G4 views, supporting unified cutoffs across imaging orientations and reducing the need for view-specific adjustment.

## 5. Study Limitations

This study utilizes a retrospective and single-center design, which may contribute to selection bias. The limited number of cases with F0 fibrosis in this cohort reflects the tertiary referral status of the center, as most biopsies are conducted for chronic liver disease. In these biopsy-selected instances, severe fibrosis or cirrhosis not associated with viral hepatitis is typically linked to conditions such as autoimmune hepatitis, primary biliary cholangitis, or metabolic-associated fatty liver disease.

Fibrosis has the potential to alter echotexture, which may influence DL measurement of steatosis. Analysis indicates that DL-based steatosis scores align closely with histologic grades of steatosis regardless of fibrosis stage (partial correlation r = 0.802, *p* < 0.001). Additionally, when controlling steatosis grades, there is a negative correlation between DL-based steatosis scores and fibrosis grades (partial correlation r = −0.281, *p* < 0.001), which may indicate that fibrosis is associated with reduced liver steatosis. These findings suggest that fibrosis does not have a significant influence on the diagnosis of steatosis.

Another limitation is that gain settings were operator-dependent, and we had to assume optimal adjustments were made during image acquisition. Image quality is a key requirement for accurate measurement. For scanners not included in the training set [18], correlation studies—such as those performed on the Philips Affiniti 70 in this study (Figure 4)—could be conducted using paired images from old and new scanners taken on the same date, allowing potential adjustments to the cutoff values (Supplementary Table S2). In addition, external validation from other institutions will be needed.

Lastly, liver fat distribution is often uneven [34]. Our approach—using a mean score derived from 3–5 separate images per view—helps minimize the impact of regional fat variability. However, more targeted studies and larger image series are needed to further optimize this strategy.

## 6. Conclusions

Despite these limitations, our results indicate that DL algorithms can objectively quantify liver steatosis from 2D ultrasound images. The high AUROC and accuracy across multiple scanners and imaging views, together with the transferability of cutoff values and validation on a newly introduced ultrasound system, support its potential for real-world clinical implementation and research use.

## Figures and Tables

**Table 1:** Demographics.

Category	NBNC	HBV or HCV	*p* Value
Case Number	390	67	
Female, n (%)	191 (49.0%)	24 (35.8%)	0.046
Male n (%)	199 (51.0%)	43 (64.2%)	
Age (year) ^†^	49.2 ± 14.0	55.3 ± 11.0	<0.001
AST (U/L) ^†^	95.9 ± 129.0	115.1 ± 207.3	NS
ALT (U/L) ^†^	180.4 ± 273.2	212.7 ± 426.1	NS
ALK (U/L) ^†^	113.8 ± 113.3	90.8 ± 55.3	NS
g-GT (U/L) ^†^	163.2 ± 232.4	144.0 ± 180.8	NS
Bilirubin (mg/dL) ^†^	2.0 ± 3.5	2.5 ± 4.1	NS
Platelet (10^9^/L) ^†^	243.4 ± 74.9	185.2 ± 64.9	<0.001
PT-INR ^‡^	1.06 ± 0.12	1.15 ± 0.25	0.006
Cholesterol (mg/dL) ^†^	200.7 ± 47.3	174.3 ± 47.7	0.003
Triglyceride (mg/dL) ^†^	168.5 ± 96.0	179.1 ± 188.3	NS
Body mass index ^†^	26.6 ± 4.6	26.3 ± 5.3	NS
Associated etiology			NS
Acute hepatitis, n (%)	23 (5.9%)	3 (4.5%)	
AIH ^§^, n (%)	46 (11.8%)	6 (9.0%)	
PBC ^¶^, n (%)	31 (7.9%)	3 (4.5%)	
Steatosis (histology)			<0.001
S0, n (%)	125 (32.1%)	26 (38.8%)	
S1, n (%)	52 (13.3%)	21 (31.3%)	
S2, n (%)	69 (17.7%)	10 (14.9%)	
S3, n (%)	144 (36.9%)	10 (14.9%)	
Fibrosis (histology) *			<0.001
F0, n (%)	61 (15.6%)	4 (6.0%)	
F1, n (%)	202 (51.8%)	22 (32.8%)	
F2, n (%)	53 (13.6%)	12 (17.9%)	
F3, n (%)	47 (12.1%)	11 (16.4%)	
F4, n (%)	27 (6.9%)	18 (26.9%)	

^†^ Mean ± standard deviation; * 5 patients without fibrosis data. Abbreviation: ^‡^ PT-INR: prothrombin time international normalized ratio; ^§^ AIH: autoimmune hepatitis; ^¶^ PBC: primary biliary cholangitis.

**Table 2:** Scanners used in this study.

Scanners	Philips		SuperSonic	Siemens	Toshiba	Hitachi	Total
	IU22	Affiniti 70	Mach 30	Acuson S2000	TUS-A300	HI VISION Preirus	
Total	41	51	5	439	46	6	588
%	7.0%	8.7%	0.9%	74.7%	7.8%	1.0%	100.0%

**Table 3:** AUROC of DL model, CAP, and BMI for hepatic steatosis classification across different ultrasound views.

View	Model	Steatosis Grade		
		Grade 0 vs. 1–3	Grade 0–1 vs. 2~3	Grade 0–2 vs. 3
		AUROC (95% CI)	AUROC (95% CI)	AUROC (95% CI)
G2	DL	0.929 (0.906–0.952)	0.927 (0.904–0.949)	0.904 (0.878–0.929)
	CAP	0.905 (0.877–0.932)	0.869 (0.839–0.898)	0.840 (0.808–0.872)
	BMI	0.817 (0.780–0.854)	0.722 (0.680–0.764)	0.692 (0.648–0.736)
G1	DL	0.936 (0.912–0.960)	0.927 (0.902–0.951)	0.915 (0.890–0.940)
	CAP	0.901 (0.870–0.933)	0.871 (0.838–0.903)	0.842 (0.807–0.877)
	BMI	0.807 (0.766–0.849)	0.734 (0.688–0.779)	0.718 (0.671–0.766)
G4	DL	0.891 (0.858–0.923)	0.914 (0.883–0.945)	0.899 (0.865–0.933)
	CAP	0.865 (0.824–0.905)	0.883 (0.847–0.920)	0.882 (0.846–0.919)
	BMI	0.810 (0.764–0.856)	0.771 (0.721–0.822)	0.759 (0.706–0.811)

**Table 4:** Performance of G2-derived DL steatosis score cut-offs applied to G1 and G4 views.

Model	Steatosis Grade	Cutoff	Sensitivity	Specificity	Accuracy
DL-G2	S3	0.55	0.825	0.825	0.825
N = 565	S2	0.44	0.861	0.870	0.865
	S1	0.26	0.866	0.863	0.865
DL-G1	S3	0.55	0.918	0.818	0.849
N = 464	S2	0.44	0.867	0.861	0.864
	S1	0.26	0.890	0.844	0.875
DL-G4	S3	0.55	0.769	0.872	0.845
N = 341	S2	0.44	0.818	0.868	0.848
	S1	0.26	0.892	0.658	0.792
CAP	S3	288	0.740	0.755	0.750
N = 565	S2	274	0.801	0.803	0.802
	S1	251	0.853	0.852	0.853

**Table 5:** Summary of recent DL studies with more than binary steatosis grades.

Reference	Tai J	Kaffas AE [30]	Jeon SK [14]	Vianna P [24]	Li B [18]	Byra M [22]	Chen JR [21]
Pub. year	Current	2025	2023	2023	2022	2021	2020
Total cases	457	403	173	199	3,569	135	41
US studies	588	403	173	199	19,513	135	164
US scanner	6 models	GE Canon	RS85 Medison	7 models	13 models	Siemens S3000	Terason M3000
Image type	Grayscale	Grayscale	Grayscale RF (TAI,TSI)	Grayscale	Grayscale	Grayscale	Grayscale
Images/Case	≥5	**-**	**-**	**-**	>= 10	4	5
Total Images	7003	96,994	**-**	7529	228,075	540	205
AOI	256 × 256	224 × 224	2 × 4 cm AOI	128 × 128	256 × 256	256 × 256	3.5 × 3.5 AOI
Gold Standard	Histology	MRI PDFF	MRI PDFF	Histology	Histology	MRI PDFF	Histology
View	3 views	Liver/kidney	RLL	3 views	4 Views	RLL and LKC	RLL
DL Model	ResNet-18	Mobile NetV3	2D CNN	VGG16	ResNet-18	ResNet-50	VGG-16
AUROC							
Grade ≥ 1	0.929	0.813	0.97	0.98	0.85–0.95	0.91	0.71
Grade ≥ 2	0.926	0.959	0.96 ^@^	0.67	0.91–0.92	0.86 ^$^	0.75
Grade ≥ 3	0.906	-	0.95 ^#^	0.66	0.87–0.93	-	0.88

@ <15% vs. ≥15%; # <25% vs. ≥25%; $ <10% vs. ≥10%. Abbreviations: AOI: Area of interest; RF: radiofrequency; RLL: Right liver lobe; LKC: liver/kidney contrast; TAI: tissue attenuation imaging; TSI: tissue scatter distribution imaging.

## Data Availability

Data is unavailable due to privacy.

## Supplementary Materials

The following supporting information can be downloaded at: https://www.mdpi.com/article/10.3390/diagnostics16020267/s1, Figure S1: ROC curves for DL steatosis score, FibroScan CAP, and BMI in diagnosing liver steatosis with the G1 view; Figure S2: ROC curves for DL steatosis score, FibroScan CAP, and BMI in diagnosing liver steatosis with the G2 view; Table S1: Cross Confusion Matrix: Comparative analysis of DL and CAP predictions across histological grades in the G2 view; Table S2: DL model performance compared between Acuson S2000 and Affiniti 70 scanners using original and adjusted cutoffs.

## Abbreviations

The following abbreviations are used in this manuscript:

AUROC	Area under receiver operating characteristic curve
AIH	Autoimmune hepatitis
CAP	Controlled attenuation parameter
DL	Deep learning
HBV	Hepatitis B virus
HBsAg	Hepatitis B surface antigen
HCV	Hepatitis C virus
MAFLD	Metabolic dysfunction-associated fatty liver disease
MRI-PDFF	Magnetic resonance imaging proton density fat fraction
PBC	Primary biliary cirrhosis
QUS	Quantitative ultrasounds
TAI	Tissue attenuation imaging
TSI	Tissue scatter-distribution imaging
