# Supplementary material for: Clinical Validation of a Deep Learning-Based 2D Ultrasound Steatosis Algorithm: Cutoff Transferability, Scanner Generalizability, and Comparison with FibroScan
Source: Diagnostics (Basel). 2026 Jan 14;16(2):267. doi: 10.3390/diagnostics16020267 (PMC12840080; doi:10.3390/diagnostics16020267)
Supplement: Supplementary file 1 [file diagnostics-16-00267-s001.zip › diagnostics-3942633-supplementary.pdf]

Supplementary

Figure S1. ROC curves for DL steatosis score, FibroScan CAP, and BMI in diagnosing liver steatosis with the G1 view.

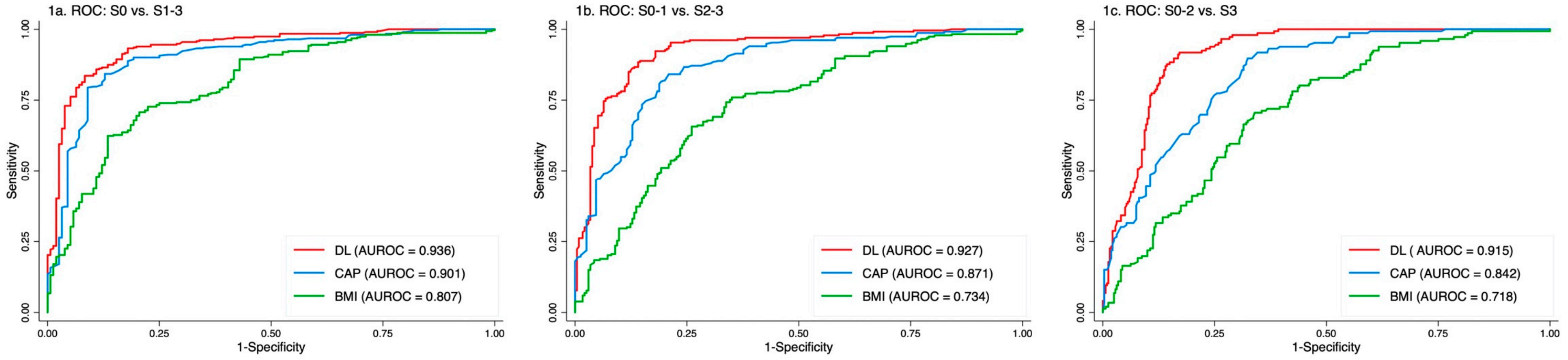

Figure S2. ROC curves for DL steatosis score, FibroScan CAP, and BMI in diagnosing liver steatosis with the G2 view.

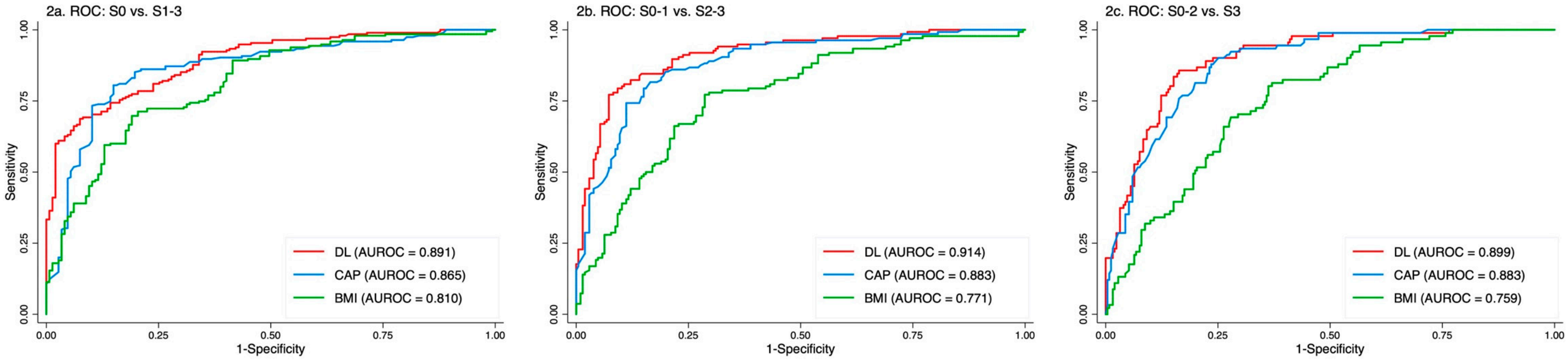

Table S1. Cross Confusion Matrix: Comparative analysis of DL and CAP predictions across histological grades in the G2 view.

Detail distribution	DL prediction	CAP prediction 0	1	2	3
Histology grade 3 vs 0-2	0	0	0	0	2
	1	2	1	1	2
	2	3	4	6	10
	3	4	8	17	117
Histology grade 2-3 vs 0-1	0	8	1	1	2
	1	6	6	3	12
	2	7	9	10	20
	3	6	13	25	152
Histology grade 1-3 vs 0	0	26	12	2	11
	1	16	15	6	23
	2	8	16	12	25
	3	6	18	26	160

Summary

	DL prediction	CAP prediction Negative	CAP prediction Positive	P value
Histology grade 3 vs 0-2	Negative	17	14	
	Positive	29	117	<0.001
Histology grade 2-3 vs 0-1	Negative	21	18	
	Positive	35	207	<0.001
Histology grade 1-3 vs 0	Negative	26	25	
	Positive	30	301	<0.001

Table S2. DL model performance compared between Acuson S2000 and Affiniti 70 scanners using original and adjusted cutoffs.

Model-view	Scanner	Steatosis grade	Cutoff	Sensitivity	Specificity	Accuracy
DL-G2 N=47	S2000	S3	0.55	0.778	0.816	0.809
		S2	0.44	0.700	0.889	0.809
		S1	0.26	0.727	0.929	0.787
	Affiniti 70	S3	0.55	1.000	0.711	0.766
		S2	0.44	0.850	0.667	0.745
		S1	0.26	1.000	0.579	0.660
DL-G1 N=46	S2000	S3	0.55	1.000	0.784	0.826
		S2	0.44	0.800	0.846	0.826
		S1	0.26	0.833	0.938	0.870
	Affiniti 70	S3	0.55	0.889	0.730	0.761
		S2	0.44	0.950	0.692	0.804
		S1	0.26	0.933	0.688	0.848
CAP N=47	FibroScan	S3	288	0.778	0.658	0.681
		S2	274	0.800	0.704	0.745
		S1	251	0.879	0.714	0.830
Adjusted cutoff system						
DL-G2 N=47	Affiniti 70	S3	0.63	0.889	0.789	0.809
		S2	0.46	0.850	0.778	0.809
		S1	0.36	0.727	0.714	0.723
DL-G1 N=46		S3	0.63	0.889	0.811	0.826
		S2	0.46	0.800	0.692	0.739
		S1	0.36	0.867	0.750	0.826

Supplementary Legends

Figure S1. ROC curves for DL steatosis score, FibroScan CAP, and BMI in diagnosing liver steatosis with the G1 view. For moderate (1b) and severe (1c) histological grades of steatosis, the DL steatosis scores showed significantly better performance than CAP.

Figure S2. ROC curves for DL steatosis score, FibroScan CAP, and BMI in diagnosing liver steatosis with the G2 view. DL steatosis score and FibroScan CAP had similar predictive ROC curves for all histological steatosis grades.

Table S1. Cross Confusion Matrix: Comparative analysis of DL and CAP predictions across histological grades in the G2 view. DL predictions demonstrate statistically significant superiority over CAP for every histological grade ($P<0.001$).

Table S2. DL model performance compared between Acuson S2000 and Affiniti 70 scanners using original and adjusted cutoffs. Affiniti 70 showed lower accuracy than S2000 for predicting liver steatosis with the original cutoff, but its accuracy improved after applying the adjusted cutoff.
